# Systematic analyses of genetic variants in chromatin interaction regions identified four novel lung cancer susceptibility loci

**DOI:** 10.7150/jca.35127

**Published:** 2020-01-01

**Authors:** Pei Ji, Dongsheng Ding, Na Qin, Cheng Wang, Meng Zhu, Yuancheng Li, Juncheng Dai, Guangfu Jin, Zhibin Hu, Hongbing Shen, Liang Chen, Hongxia Ma

**Affiliations:** 1 Department of Epidemiology, Center for Global Health, School of Public Health, Nanjing Medical University, Nanjing, China.; 2 Department of Bioinformatics, School of Basic Medical Sciences, Nanjing Medical University, Nanjing, China.; 3 Jiangsu Key Lab of Cancer Biomarkers, Prevention and Treatment, Collaborative Innovation Center for Cancer Medicine, Nanjing Medical University, Nanjing, China.; 4 Department of Thoracic Surgery, The First Affiliated Hospital of Nanjing Medical University, Nanjing, China.

**Keywords:** lung cancer, single-nucleotide polymorphisms (SNPs), chromatin interactions, Genome-wide association studies (GWAS), expression quantitative trait loci.

## Abstract

Genome-wide association studies (GWAS) have reported 45 single-nucleotide polymorphisms (SNPs) that may contribute to the susceptibility of lung cancer, with the majority in non-coding regions. However, no study has ever systematically evaluated the association between SNPs in physical chromatin interaction regions and lung cancer risk. In this study, we integrated the chromatin interaction information (Hi-C data) of lung cancer cell line and conducted a meta-analysis with two Asian GWASs (7,127 cases and 6,818 controls) to evaluate the association of potentially functional SNPs in chromatin interaction regions with lung cancer risk. We identified four novel lung cancer susceptibility loci located at 1q21.1 (rs17160062, *P*=4.00×10^-6^), 2p23.3 (rs670343, *P*=4.87×10^-7^), 2p15 (rs9309336, *P*=3.24×10^-6^) and 17q21.2 (rs9252, *P*=1.51×10^-5^) that were significantly associated with lung cancer risk after correction for multiple tests. Functional annotation result indicated that these SNPs may contribute to the development of lung cancer by affecting the availability of transcription factor binding sites. The HaploReg analysis suggested that rs9309336 may affect binding motif of transcription factor Foxp1. Expression quantitative trait loci analysis revealed that rs9309336 and rs17160062 could regulate the expressions of cancer-related genes (*PUS10* and* CHD1L*). Our results revealed that variants in chromatin interaction regions could contribute to the development of lung cancer by regulating the expression of target genes, which providing novel implications for the understanding of functional variants in the development of lung cancer.

## Introduction

Lung cancer is the leading cause of cancer-related deaths all over the world 1. The estimated incidence of lung cancer in Asian is more than 1.04 million and the mortality is 0.94 million according to the international project GLOBOCAN 2012 2. Cigarette smoking is considered as the major cause of lung cancer; however, less than 20% of smokers develop lung cancer. Inherited genetic factor may also play an important role in the development of lung cancer 3,4.

Recently, genome-wide association studies (GWASs) have become the most widely used technique to identify susceptibility loci associated with diseases. For lung cancer, a total of 45 genomic loci have been identified that contribute to lung cancer risk 5. However, most of these SNPs lie in noncoding regions of the genome with the causal ones remain unknown 6-9. Some studies have identified that gene expression is often influenced by regulatory elements in kilobases (kb) to megabases (Mb) upstream or downstream 10 which are brought in close proximity to one another through chromatin interactions, defining the chromatin architecture of the genome 11. These findings and the development of high-throughput technology enable researchers to pinpoint causal variants and genes. For example, by combining DNA proximity ligation with high-throughput sequencing, Hi-C technology have allowed the capture of genome-wide chromatin interactions 12,13, which highlight the importance of chromatin interactions between regulatory elements and their target genes in GWAS interpretation. Recently, several studies have performed Hi-C experiments in many cancers 14,15, which provide important support to systematically investigate causal variants in regulatory elements and the risk of human cancer. By integrative analysis of the expression quantitative trait loci (eQTL) and Hi-C data, we were able to define risk SNPs in regulatory elements which may contribute to the risk of cancer by modulating the expression of candidate genes. For example, Li Q *et al.* conducted a eQTL-based analysis and identified that several breast cancer risk SNPs in enhancers could contribute to the risk of breast cancer by modulating the expression of critical cancer-associated genes 16. Therefore, the integration of chromatin interaction information and eQTL analyses can efficiently identify candidate causal loci and genes associated with the development of cancer. These findings provide further support for the hypothesis that chromatin interaction links SNPs in regulatory elements to target genes, thus affecting tumorigenesis by modulating target gene expression. It was therefore possible to delineate SNPs with evidence for being causative on the basis of their profiles for chromatin state and eQTL analysis.

In this study, we conducted a GWAS meta-analysis of 7,127 lung cancers and 6,818 healthy controls with Asian ancestry (GWAS from Nanjing Medical University (NJMU) and Female Lung Cancer Consortium in Asia (FLCCA)). By integrating whole-genome chromatin interaction maps and eQTL analysis, we systematically investigated the functional variants and candidate genes that contribute to the susceptibility of lung cancer risk.

## Materials and methods

### Study populations

Two previously published lung cancer GWAS data sets were included in this study, including one from NJMU and one from FLCCA. NJMU GWAS was consistent of 2,383 lung cancers and 3,160 healthy controls from the Han Chinese population as previously reported 17. Briefly, The cases were newly diagnosed and histopathologically or cytologically confirmed lung cancer by at least two local pathologists. We enrolled patients with primary lung cancer and previously untreated. Patients with any prior cancer history were not recruited. All cancer-free controls were selected from a community-based screening program for non-infectious disease and frequency-matched for age, gender to the lung cancer cases. FLCCA data was obtained from the NCBI database of Genotypes and Phenotypes (dbGaP) 18, which included 4,922 lung cancer cases and 3,959 controls from 14 lung cancer studies in East Asia (https://www.ncbi.nlm.nih.gov/projects/gap/cgi-bin/study.cgi?study_id=phs000716.v1.p1). All samples from the FLCCA GWAS were females and non-smokers and details were described in a previous study 17. All lung cancer cases were histologically confirmed. Each study was approved by local institutional review board and all study participants provided informed consent prior to participation.

### Quality control and imputation of GWAS data

The subjects from the NJMU GWAS and FLCCA GWAS were separately genotyped using the Affymetrix Genome-Wide Human SNP Array 6.0 and the Illumina Human660W-Quad v1.0 DNA Analysis BeadChip platform. We performed a stringent quality control procedure for both NJMU and FLCCA GWAS data. Individuals satisfied any of the following criteria will be removed: (1) call rate <95%; (2) gender discordance; (3) samples with familial relationships; (4) an extreme heterozygosity rate; (5) outliers according to a principal component analysis (PCA). For the FLCCA study, we also excluded the overlapped subjects between FLCCA GWAS and NJMU GWAS. SNPs with call rate < 95%, Hardy-Weinberg equilibrium (HWE) *P*-value< 1×10^-6^ or a minor allele frequency (MAF) < 5% were excluded from the following analysis. Imputation was conducted by using the IMPUTE2 software, and the 1000 Genomes Project Phase II data (version 3) was set as the reference. We conducted the pre-phasing strategy with SHAPEIT (version 1), and the phased haplotypes were set as the input for IMPUTE2. A post-imputation QC was further conducted to filter unqualified SNPs, and only SNPs with call rate >95%, MAF >0.05, *P*>0.05 for HWE in all samples and high imputation quality (Imputation score≥0.8) were included. Finally, 7,569,840 SNPs in 2,331 lung cancers and 3,077 controls from NJMU GWAS and 7,608,635 SNPs in 4,796 lung cancers and 3,741 controls from FLCCA GWAS were remained for further analysis.

### Selection of genetic variants

We obtained the Hi-C data of A549 cell line from the Encyclopedia of DNA Elements (ENCODE) Consortium resources (https://www.encodeproject.org/experiments/ENCSR662QKG/) as it was the only one lung-related cell line with long range chromatin interactions data available. The genomic coordinates were lifted over from NCBI human genome build 38 to build 37 using the UCSC LiftOver tool. A total of 363,334 SNPs in 25,666 chromatin interaction regions from both FLCCA and NJMU GWASs were included in the analysis.

To further filter SNPs with less functional evidence, we performed functional annotation with RegulomeDB (http://www.regulomedb.org/) 19 and retained 36,249 potentially functional SNPs (RegulomeDB score ≤ 3) for further analysis. Additionally, we used PriorityPruner software (version 0.1.4) and dropped 12,506 SNPs from further analysis because they are in high linkage disequilibrium (R^2^≥0.8) with the remaining SNPs. The detailed workflow chart was shown in Fig. S1.

### Functional annotations of promising SNPs

We performed functional annotation based on ENCODE project from UCSC Genome Bioinformatics website (http://genome.ucsc.edu/) to explore the potential function of the candidate SNPs. Chromatin biofeatures including transcription factor bites of 161 transcription factors from ENCODE Factorbook Motifs, DNaseI Hypersensitivity Clusters in 125 cell types from ENCODE and histone modifications of epigenetic markers H3K4me1, H3K4me3, H3K9ac and H3K27ac on NHLF andA549 cell lines. We used HaploReg v4.1 ( https://pubs.broadinstitute.org/mammals/haploreg/haploreg.php ) to evaluate the effect of identified SNPs on transcription factor binding site motifs 20.

### eQTL and differential expressed analyses

Our study previously performed whole-genome sequencing (WGS) and RNA-sequencing (RNA-seq) on 90 tumor/blood/adjacent pairs of lung cancer patients from China 21. The expression data was quantified as FPKM. Paired Wilcoxon rank sum test was performed to evaluate the differential expression of target genes in 90 tumor/adjacent pairs. A linear regression model was used to perform the eQTL analysis with adjustment for age, gender, smoking status and PCAs in 90 adjacent samples. The expression was log2-transformed. Characteristics and clinical features of the patients are shown in Table S1.

### Statistical analysis

Differences in the distribution of baseline characteristics including continuous variable like age was compared by Welch's t-test and classified variables like gender and smoking status were evaluated by χ2 test. Multivariate logistic regression model was used to evaluate the association of SNPs with lung cancer with adjustment for age, sex, smoking status and principal components. Fixed-effects meta-analysis was used to combine individual association estimates from FLCCA and Nanjing GWAS. The BH-FDR procedure (Benjamini-Hochberg false discovery rate) method was applied to account for multiple comparisons. *P* values were two sides and corrected *P (P*_FDR-BH_*)* <0.05 was considered as statistically significant. Meanwhile, these SNPs have the same direction of effect in two GWASs and *P*_heterogeneity_> 0.05 in meta-analysis. These statistical analyses were performed with R software (version 3.3.2).

## Results

### Association between genetic variants in chromatin interaction regions and lung cancer risk

A total of 7,127 cases and 6,818 controls were enrolled in this study, and the baseline characteristics and clinical features of the participants were shown in Table S2. The NJMU GWAS was consisted of 2,331 cases and 3,077 controls, while the FLCCA GWAS included 4,796 cases and 3,741 controls. 36,249 SNPs located in the chromatin interaction regions with RegulomeDB score ≤ 3 were included for further analysis. We used LD (linkage disequilibrium) analyses to select those SNPs with lowest *P* values among multiple SNPs in high LD (R^2^≥ 0.8) and performed logistic regression analysis. We then carried out a meta-analysis based on the results of NJMU and FLCCA GWAS. The result revealed that five SNPs reached the significance level (*P*_FDR_<0.05), including rs4946258 (adjusted OR=0.88, *P*=4.16×10^-7^), rs670343 (adjusted OR=0.88, *P*=4.87×10^-7^), rs9309336 (adjusted OR=0.89, *P*=3.24×10^-6^), rs17160062 (adjusted OR=0.84, *P*=4.00×10^-6^) and rs9252 (adjusted OR=0.85, *P*=1.51×10^-5^) (Table 1). Among those, rs4946258 was in strong linkage disequilibrium with a previously reported susceptibility SNP rs9387478 (R^2^ = 0.777) 18. The genotype distributions of four novel SNPs and their association with lung cancer risk were shown in Table 1.

Stratified analyses according to age, gender, smoking status and histology subtypes were further used to examine the associations of the four novel SNPs and lung cancer risk. Interestingly, we identified that the protective effect of rs9309336 was significantly stronger in smokers than that in non-smokers (*P*_heterogeneity_ = 0.033) (Table 2).

### Functional annotation of four novel SNPs

We then performed functional annotation of four novel variants with ENCODE regulatory data downloaded from the UCSC Genome Bioinformatics website (http://genome.ucsc.edu/) to estimate the potential function. Rs670343 at 2p23.3 resided in an enhancer element in NHLF and A549 cell lines, while rs9309336 at 2p15 fell into the promoter histone marks (Fig. S2). Rs17160062 was located in a genomic region with transcription factor (CEBPB) binding loci (ChIP-seq peaks). Rs9252 at 17q21.2 was located in the DNaseI Hypersensitivity cluster and overlapped with H3K4me1 modified region on both NHLF and A549 cell lines (Fig. S2). Moreover, the HaploReg analysis showed that rs9309336 affect binding motifs of important transcription factors such as Foxp1. Rs9309336 was highly enriched for promoters and DNase hypersensitive regions across multiple cell lines.

**Fig 1:**
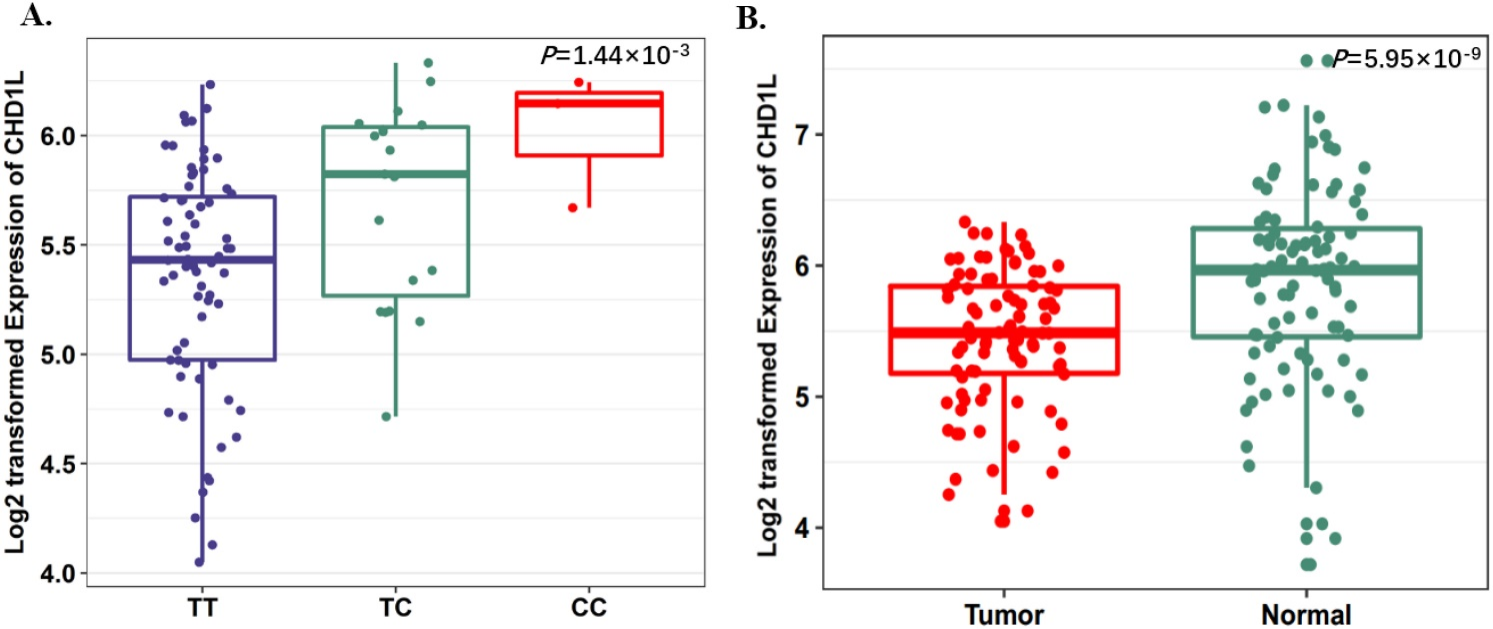
SNP rs17160062 as a possible eQTL for *CHD1L*.** A,** The boxplot shows the associations between genotypes of rs17160062 and *CHD1L* expression in 90 normal lung tissue from our previous study. The* P*-value was derived from linear regression analysis. **B,** The boxplot shows the expression level of *CHD1L* was increased in 90 tumor tissues compared with paired adjacent samples. The *P*-value was derived from Wilcoxon rank sum tests. Expression of *CHD1L* were log2 transformed. The boxplot displays the first and third quartiles (top and bottom of the boxes), the median (band inside the boxes), and the lowest and highest point within 1.5 times the interquartile range of the lower and higher quartiles (whiskers).

**Fig 2:**
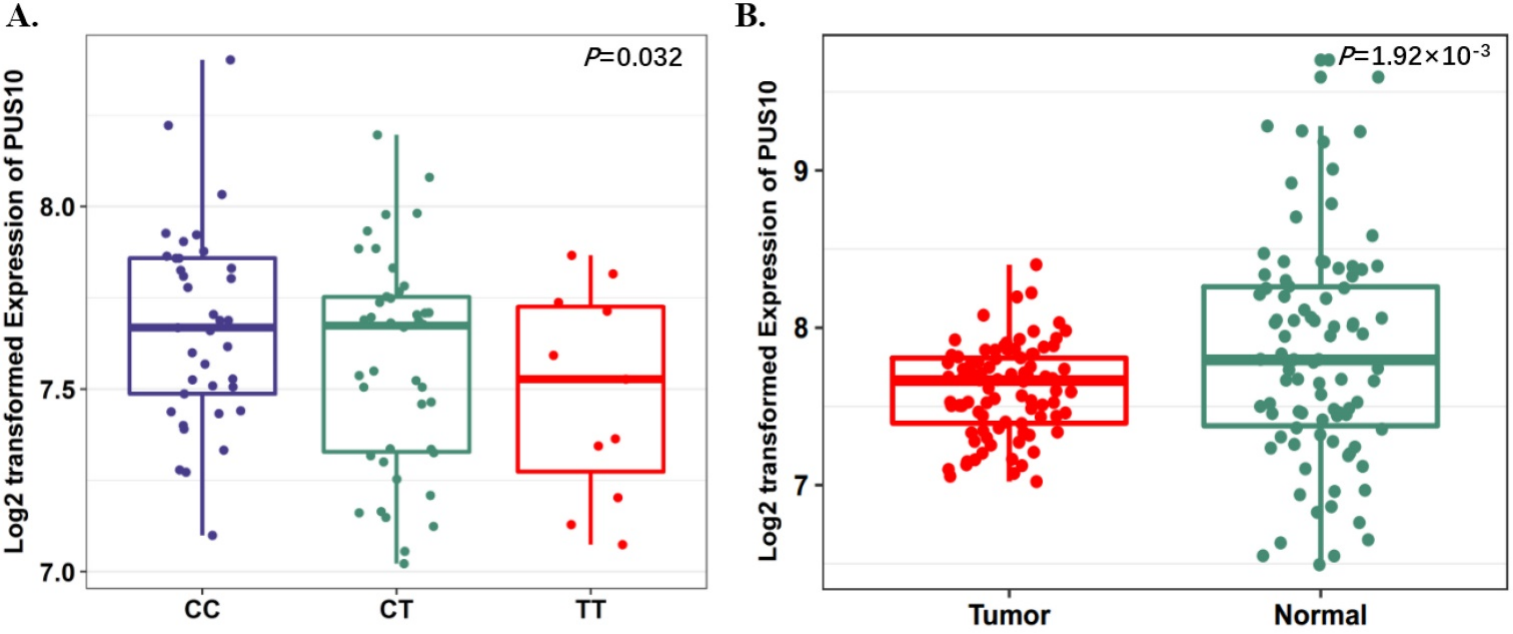
Expression quantitative trait loci (eQTL) analyses of rs9309336 with* PUS10* mRNA expression levels. **A,** The boxplot shows the associations between genotypes of rs9309336 and *PUS10* expression in 90 normal lung tissue from our previous study. The* P*-value was derived from linear regression analysis. **B,** The boxplot shows the expression level of *PUS10* was increased in 90 tumor tissues compared with paired adjacent samples. The *P*-value was derived from Wilcoxon rank sum tests. Expression of *PUS10* were log2 transformed. The boxplot displays the first and third quartiles (top and bottom of the boxes), the median (band inside the boxes), and the lowest and highest point within 1.5 times the interquartile range of the lower and higher quartiles (whiskers).

To further evaluate the function of the candidate genes, we conducted differential expression analyses and eQTL analysis with data from our previous study. The results showed that rs17160062 at 1q21.1 was a cis-eQTL for *CHD1L*, and the C-allele was associated with an increased expression of *CHD1L* in adjacent lung tissues (*P*=1.44×10^-3^, Fig. 1A). The expression level of *CHD1L* was significantly higher in lung tumor tissues than that in adjacent normal controls (*P*= 5.95×10^-9^, Fig. 1B). Additionally, the T allele of rs9309336 was significantly associated with a decreased *PUS10* expression in adjacent lung tissues (*P*= 0.032, Fig. 2A), and *PUS10* has a significantly increased expression in lung tumor tissues than that in adjacent tissues (*P*= 1.92×10^-3^, Fig. 2B).

## Discussion

To date, GWAS have identified 45 risk loci for lung cancer 5; however, these variants explain only about 15.17% of the heritability of lung cancer 22. Therefore, it is still a challenge to identify more susceptibility loci. Recently, Du M *et.al.* found that genetic variants in the regulatory elements could affect the risk of prostate cancer by regulating target gene expression through chromatin interactions 23, which revealing that leveraging the physical interaction information may help to prioritize the GWAS signals of human cancers. In this present study, we integrated the chromatin interaction information of lung cancer cell line and two GWA studies in Asian population to systematically investigate the association of variants located in the chromatin interaction regions with lung cancer risk. We found four novel SNPs (rs670343, rs9309336, rs17160062, rs9252) were significantly associated with the risk of lung cancer; and functional annotation indicated rs17160062 and rs9309336 might affect transcriptional regulation and the expression of certain important genes.

Rs17160062 was an Asian ancestry-specific causal variant of lung cancer (MAF_Asain_=0.09, MAF_European_=0.008), which located in the fifth intron of *CHD1L* (chromodomain helicase/ATPase DNA binding protein 1-like gene). Functional annotation suggested the potential regulatory role of rs17160062 and C allele of rs17160062 was associated with an increased expression of *CHD1L*, the expression of which was significantly higher in tumor samples compared with that in the adjacent tissues. *CHD1L* is a newly identified oncogene that is amplified in many solid tumors 24,25. Functional studies suggested that *CHD1L* plays an oncogenic role in the tumorigenesis of hepatocellular carcinoma 24, colorectal carcinoma 26 and other tumors through unleashed cell proliferation, G1/S transition and inhibition of apoptosis^27^. Chen M *et.al* found that *CHD1L* could facilitate DNA synthesis and G1/S transition through the up-regulation of Cyclin A, Cyclin D1, Cyclin E, CDK2, and CDK4, and down-regulation of Rb, p27(Kip1), and p53 in a transgenic mouse model 28. In this study, we speculated that the variant of rs17160062 may play a role during the process of lung carcinogenesis through up-regulating the expression level of *CHD1L* and affecting the modification of chromatin structure.

The eQTL analysis also showed that the variant genotypes of rs9309336 were associated with a decreased expression of *PUS10* (pseudouridylate synthase 10). Rs9309336 was 500kb upstream of *PUS10* and likely to modify the binding affinity of the promoter to transcription factors thereby regulating gene expression. *PUS10* is a member of pseudouridylate synthases, and is required for the TRAIL-induced apoptotic signal to progress through the intrinsic pathway by RNAi-based phenotypic screening 29. We supposed that rs9309336 may interfere with the expression of *PUS10* and reduce the sensitivity of tumor cells to TRAIL, which in turn promoting tumor cells' immortality and the occurrence of lung cancer. The result of functional annotation revealed that rs9309336 fell into the promoter histone marks and may affect binding motifs of transcription factor Foxp1. Foxp1 is a member of the broadly expressed Foxp subset of “forkhead” (Fox) transcription factors and has emerged as an important transcription factor in different organs such as lung, where it influences the balance between proliferation and differentiation 30. Foxp1 plays a critical role in malignancy and it may serve as a tumor suppressor or oncogene in different cancers 31, 32.

Rs9252 was located in the 3'-untranslated region (3'-UTR) of *PTRF*(polymerase I and transcript release factor) which plays an important role in suppressing the progression of human tumors 33,34. The overexpression of *PTRF* reduces the cell migration ability and the knockdown of *PTRF* promotes cell migration and invasion 35. Several studies have revealed that the expression of *PTRF* was down-regulated in breast cancer 36, prostate cancer 37 and lung cancer 38. Biochemical studies showed that overexpression of *PTRF* led to the suppression of the* AKT/mTOR* pathway and regulated cellular processes including survival, proliferation, growth, metabolism and metastasis 39. rs9252 showed promoter histone marks and was located in the DNase Hypersensitivity cluster. We speculated that rs9252 may contribute to the risk of lung cancer patients by influencing the function of *PTRF*. However, the molecular mechanisms of the progress remain unclear.

However, several limitations to our study need to be acknowledged. First, no other lung cancer GWAS in Asian populations was available in dbGap, which may cap the meta sample sizes. Second, main findings in this this study were not validated by an independent cohort, partly due to the limited studies we can reach in dbGAP, and may yield detriment to the robust of the results.

Overall, our study systematically evaluated the association of variants in chromatin interaction regions with the risk of lung cancer. The identification of four novel variants may provide a new insight into the mechanism of regulatory roles of functional variants in chromatin interaction regions during the development of tumors. Further studies for biological mechanism of these chromatin interactions will reveal the regulatory role of identified loci in the onset or progression of human cancers.

## Supplementary Material

Supplementary figures and tables.

## Figures and Tables

**Table 1:** Summary of associated variants identified by the meta-analysis.

Position	Locus	SNP	Allele^a^	NJMU(n=5408)			FLCCA(n=8537)			Meta-analysis					RegulomeDB Score^d^
				EAF^b^	BETA	*P*	EAF^b^	BETA	*P*	EAF^b^	BETA	*P*	*P*_FDR-BH_	*P*_het_^c^	
chr1:146731367	1q21.1	rs17160062	C/T	0.12	0.15	1.55E-02	0.12	0.18	7.95E-05	0.12	0.17	4.00E-06	8.63E-03	0.687	3a
chr2:25744731	2p23.3	rs670343	A/G	0.55	-0.17	3.87E-05	0.52	-0.10	1.26E-03	0.53	-0.13	4.87E-07	1.65E-03	0.156	3a
chr2:61763165	2p15	rs9309336	C/T	0.6	0.13	3.60E-03	0.61	0.12	2.74E-04	0.61	0.12	3.24E-06	7.69E-03	0.833	3a
chr6:117803538	6q22.1	rs4946258	T/C	0.45	-0.1	2.65E-02	0.44	-0.15	3.35E-06	0.45	-0.13	4.16E-07	1.65E-03	0.339	1b
chr17:40554849	17q21.2	rs9252	A/G	0.14	-0.21	1.74E-03	0.14	-0.14	1.98E-03	0.14	-0.16	1.51E-05	2.98E-02	0.424	2b

^a^ Effect allele/other allele.^ b^ Effect allele frequency.^c^ P value for heterogeneity. ^d^ Regulome DB score is a kind of functional prediction scores that evaluate SNPs functionality based upon experimental data, such as its existence in a DNAaseI hypersensitive site or transcription factor binding site.

**Table 2:** Stratified analyses of association between identified variants and lung cancer.

Variables	rs670343				*Phet^b^*	rs9309336				*Phet^b^*
	Case AA/AG/GG	Control AA/AG/GG	Adjusted OR ^a^ (95%CI)	*P^a^*		Case CC/CT/TT	Control CC/CT/TT	Adjusted OR ^a^ (95%CI)	*P^a^*	
Age, yr										
≤60	271/571/300	446/743/332	1.23 (1.10-1.39)	4.74E-04	0.467	466/532/144	544/749/228	0.86 (0.76-0.97)	1.27E-02	0.569
>60	327/602/260	499/752/305	1.16 (1.03-1.30)	0.014		456/552/181	552/760/244	0.90 (0.80-1.02)	0.088	
Sex										
Females	151/312/157	312/469/210	1.27 (1.08-1.50)	3.72E-03	0.368	254/278/88	363/494/134	0.95 (0.80-1.12)	0.514	0.348
Males	447/861/403	633/1026/427	1.17 (1.06-1.28)	1.93E-03		668/806/237	733/1015/338	0.86 (0.78-0.95)	3.29E-03	
Smoking										
Never	210/409/206	529/863/376	1.17 (1.03-1.33)	0.019	0.571	316/396/113	657/859/252	0.99 (0.87-1.13)	0.892	0.033
Ever	388/764/354	416/632/261	1.23 (1.10-1.36)	2.09E-04		606/688/212	439/650/220	0.82 (0.73-0.92)	4.17E-04	
Histology										
Squamous cell carcinoma	229/419/174	945/1495/637	1.10 (0.97-1.24)	0.149	0.129	343/367/112	1096/1509/472	0.78 (0.68-0.88)	1.12E-04	0.098
Adenocarcinoma	321/653/330	945/1495/637	1.25 (1.13-1.38)	8.00E-06		503/610/191	1096/1509/472	0.93 (0.84-1.03)	0.143	
Other^c^	48/101/56	945/1495/637	1.36 (1.11-1.67)	3.41E-03		76/107/22	1096/1509/472	0.84 (0.68-1.05)	0.120	

^a^ Derived from additive model using logistic regression analysis with an adjustment for age, sex, smoking status and PCA. ^b^ *P* for heterogeneity test based on χ2-based Q test.

## Abbreviations

GWAS: Genome-wide association studies
SNP: single-nucleotide polymorphism
eQTL: expression quantitative trait loci
NJMU: Nanjing Medical University
FLCCA: Female Lung Cancer Consortium in Asia
dbGaP: database of Genotypes and Phenotypes
HWE: Hardy-Weinberg equilibrium
MAF: minor allele frequency
ENCODE: Encyclopedia of DNA Elements
WGS: whole-genome sequencing
RNA-seq: RNA-sequencing
