# Supplementary material for: Systematic analyses of genetic variants in chromatin interaction regions identified four novel lung cancer susceptibility loci
Source: J Cancer. 2020 Jan 1;11(5):1075–81. doi: 10.7150/jca.35127 (PMC6959073; doi:10.7150/jca.35127)
Supplement: Supplementary file 1 — Supplementary figures and tables. [file jcav11p1075s1.pdf]

Systematic analyses of genetic variants in chromatin interaction regions identified four novel lung cancer susceptibility loci

Pei Ji [1]†, Dongsheng Ding [1]†, Na Qin [1]†, Cheng Wang [1, 2, 3], Meng Zhu [1, 3], Yuancheng Li [1], Juncheng Dai [1, 3], Guangfu Jin [1, 3], Zhibin Hu [1, 3], Hongbing Shen [1, 3], Liang Chen [4*], Hongxia Ma [1,3*]

[1] Department of Epidemiology, Center for Global Health, School of Public Health, Nanjing Medical University, Nanjing, China.

[2] Department of Bioinformatics, School of Basic Medical Sciences, Nanjing Medical University, Nanjing, China.

[3] Jiangsu Key Lab of Cancer Biomarkers, Prevention and Treatment, Collaborative Innovation Center for Cancer Medicine, Nanjing Medical University, Nanjing, China.

[4] Department of Thoracic Surgery, The First Affiliated Hospital of Nanjing Medical University, Nanjing, China.

Table S1. Patients' baseline characteristics in RNA-seq data.

Variables	LC patients (n=90)
**Age**	
≤ 60	41(45.6)
>60	49(54.4)
**Gender**	
Female	26(28.9)
Male	64(71.1)
**Smoking status**	
Never	40(44.4)
Ever	50(55.6)
**Histological cell type**	
Squamous cell carcinoma	35(38.9)
Adenocarcinoma	55(61.1)

Table S2. Distributions of characteristics in NJMU and FLCCA lung cancer GWAS cases and controls

Variables	NJMU-GWAS		FLCCA-GWAS		Overall	
	Case N (%)	Control N (%)	Case N (%)	Control N (%)	Case N (%)	Control N (%)
**All**	2331(100)	3077(100)	4796(100)	3741(100)	7127(100)	6818(100)
**Age**						
≤ 60	1142(48.99)	1521(49.43)	2408(50.21)	2023(54.08)	3306(46.39)	3266(47.90)
> 60	1189(51.01)	1556(50.57)	2388(49.79)	1718(45.92)	3821(53.61)	3552(52.10)
**Gender**						
Male	1711(73.40)	2086(67.79)	n/a	n/a	1711(24.01)	2086(30.60)
Female	620(26.60)	991(32.21)	4796(100)	3741(100)	5416(75.99)	4732(69.40)
**Smoking**						
Never	825(35.39)	1768(57.46)	4796(100)	3741(100)	5621(78.87)	5509(49.50)
Ever	1506(64.61)	1309(42.54)	n/a	n/a	1506(21.13)	1309(42.54)
**Histology**						
Squamous cell carcinoma	822(35.26)	n/a	660(13.76)	n/a	1482(20.79)	n/a
Adenocarcinoma	1304(55.94)	n/a	3469(72.33)	n/a	4773(66.97)	n/a
Other[a]	205(8.80)	n/a	667(13.91)	n/a	872(12.24)	n/a

[a] : Other histological types include small cell lung cancer, large cell lung cancer and mixed cell lung cancer.

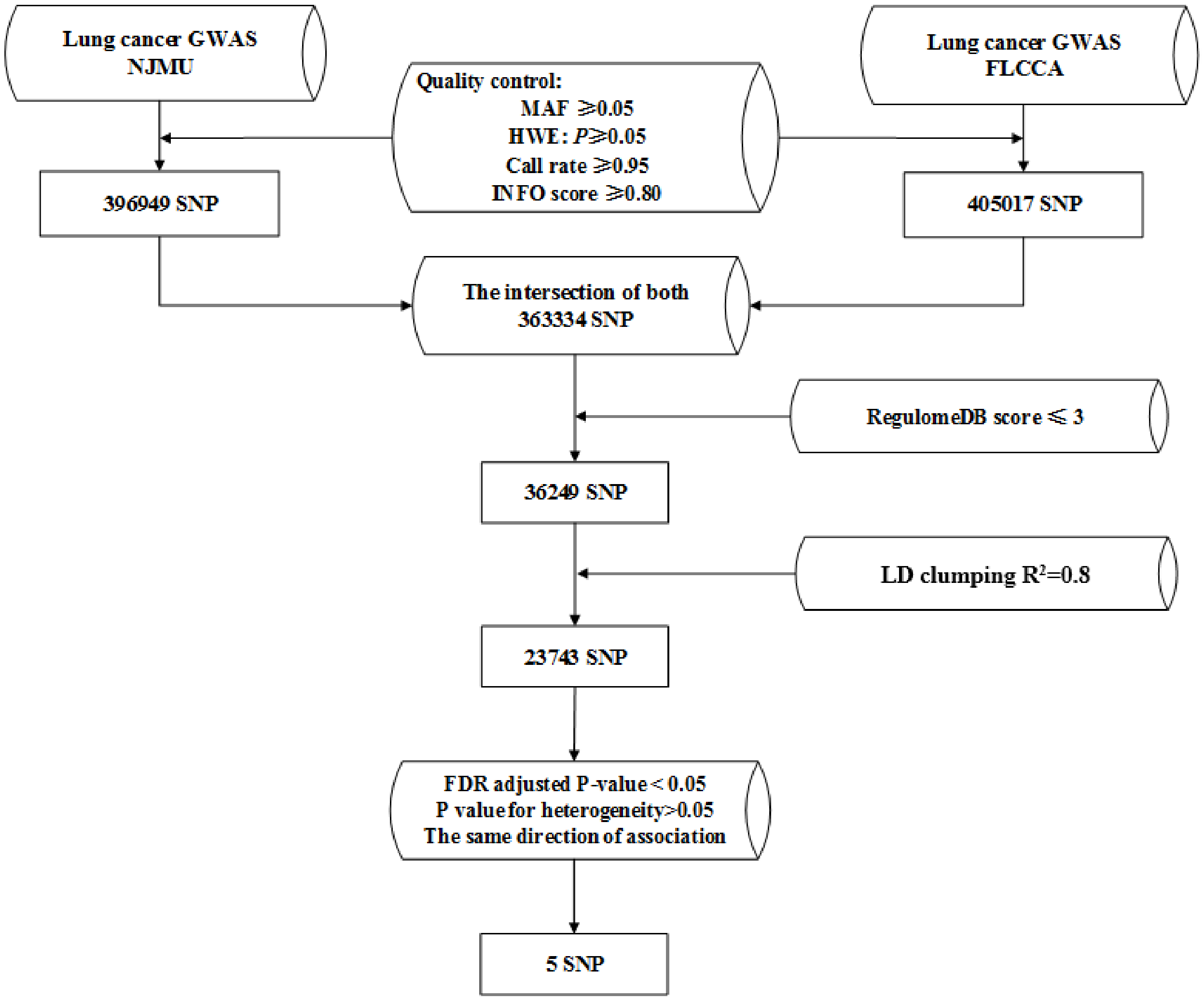

Figure S1. The workflow of SNPs screening based on two Asian GWAS data and meta-analysis.

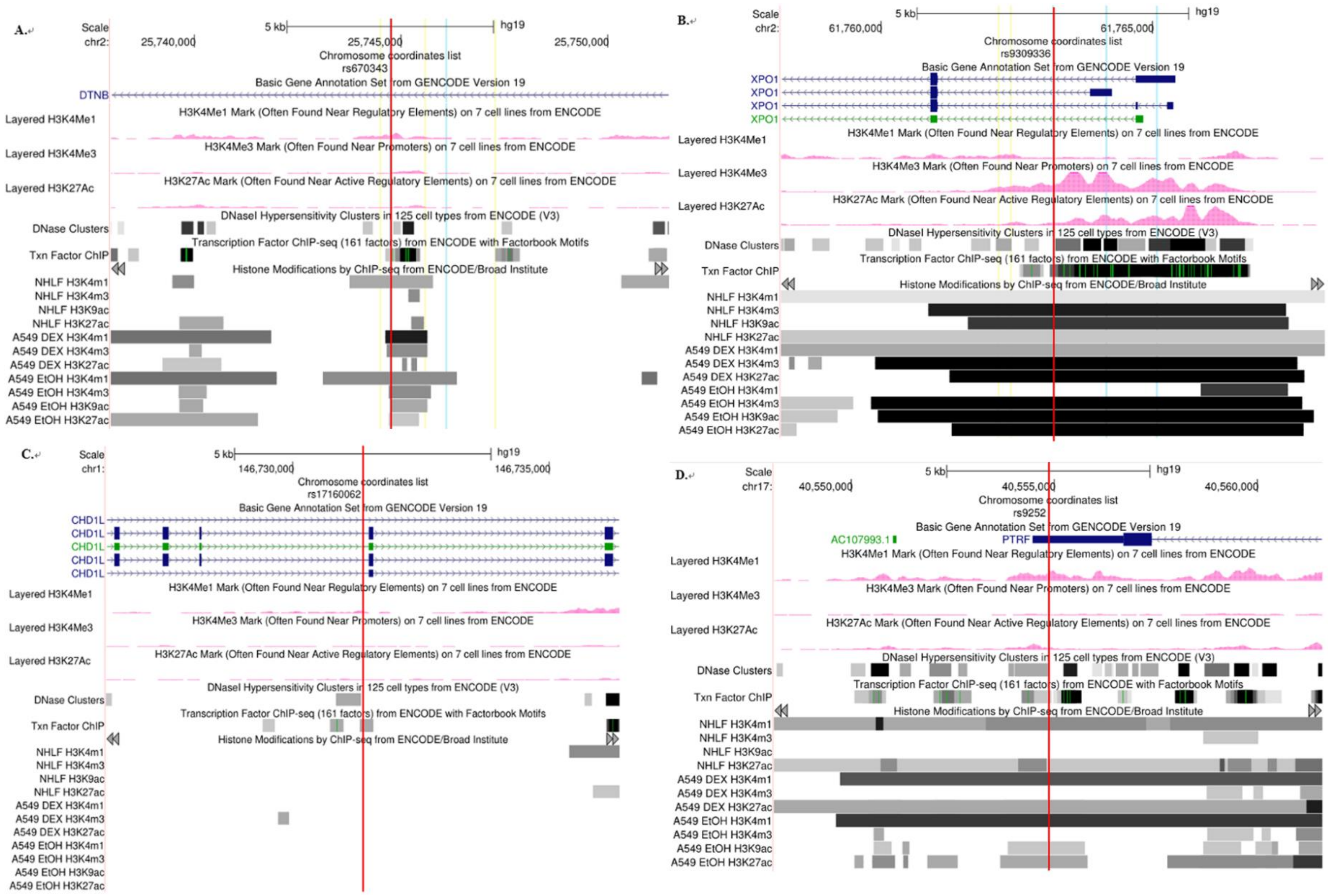

A.
Scale
chr2:
5 kb
hg19
25,740,000
25,745,000
25,750,000
Chromosome coordinates list
rs670343
Basic Gene Annotation Set from GENCODE Version 19
DTNB
Layered H3K4Me1
H3K4Me1 Mark (Often Found Near Regulatory Elements) on 7 cell lines from ENCODE
Layered H3K4Me3
H3K4Me3 Mark (Often Found Near Promoters) on 7 cell lines from ENCODE
Layered H3K27Ac
H3K27Ac Mark (Often Found Near Active Regulatory Elements) on 7 cell lines from ENCODE
DNase Clusters
DNaseI Hypersensitivity Clusters in 125 cell types from ENCODE (V3)
Txn Factor ChIP
Transcription Factor ChIP-seq (161 factors) from ENCODE with Factorbook Motifs
Histone Modifications by ChIP-seq from ENCODE/Broad Institute
NHLF H3K4m1
NHLF H3K4m3
NHLF H3K9ac
NHLF H3K27ac
A549 DEX H3K4m1
A549 DEX H3K4m3
A549 DEX H3K27ac
A549 EtOH H3K4m1
A549 EtOH H3K4m3
A549 EtOH H3K9ac
A549 EtOH H3K27ac
B.
Scale
chr2:
5 kb
hg19
61,760,000
61,765,000
Chromosome coordinates list
rs9309336
Basic Gene Annotation Set from GENCODE Version 19
XPO1
XPO1
XPO1
XPO1
Layered H3K4Me1
H3K4Me1 Mark (Often Found Near Regulatory Elements) on 7 cell lines from ENCODE
Layered H3K4Me3
H3K4Me3 Mark (Often Found Near Promoters) on 7 cell lines from ENCODE
Layered H3K27Ac
H3K27Ac Mark (Often Found Near Active Regulatory Elements) on 7 cell lines from ENCODE
DNase Clusters
DNaseI Hypersensitivity Clusters in 125 cell types from ENCODE (V3)
Txn Factor ChIP
Transcription Factor ChIP-seq (161 factors) from ENCODE with Factorbook Motifs
Histone Modifications by ChIP-seq from ENCODE/Broad Institute
NHLF H3K4m1
NHLF H3K4m3
NHLF H3K9ac
NHLF H3K27ac
A549 DEX H3K4m1
A549 DEX H3K4m3
A549 DEX H3K27ac
A549 EtOH H3K4m1
A549 EtOH H3K4m3
A549 EtOH H3K9ac
A549 EtOH H3K27ac
C.
Scale
chr1:
5 kb
hg19
146,730,000
146,735,000
Chromosome coordinates list
rs17160062
Basic Gene Annotation Set from GENCODE Version 19
CHD1L
CHD1L
CHD1L
CHD1L
CHD1L
Layered H3K4Me1
H3K4Me1 Mark (Often Found Near Regulatory Elements) on 7 cell lines from ENCODE
Layered H3K4Me3
H3K4Me3 Mark (Often Found Near Promoters) on 7 cell lines from ENCODE
Layered H3K27Ac
H3K27Ac Mark (Often Found Near Active Regulatory Elements) on 7 cell lines from ENCODE
DNase Clusters
DNaseI Hypersensitivity Clusters in 125 cell types from ENCODE (V3)
Txn Factor ChIP
Transcription Factor ChIP-seq (161 factors) from ENCODE with Factorbook Motifs
Histone Modifications by ChIP-seq from ENCODE/Broad Institute
NHLF H3K4m1
NHLF H3K4m3
NHLF H3K9ac
NHLF H3K27ac
A549 DEX H3K4m1
A549 DEX H3K4m3
A549 DEX H3K27ac
A549 EtOH H3K4m1
A549 EtOH H3K4m3
A549 EtOH H3K9ac
A549 EtOH H3K27ac
D.
Scale
chr17:
5 kb
hg19
40,550,000
40,555,000
40,560,000
Chromosome coordinates list
rs9252
Basic Gene Annotation Set from GENCODE Version 19
AC107993.1
PTRF
Layered H3K4Me1
H3K4Me1 Mark (Often Found Near Regulatory Elements) on 7 cell lines from ENCODE
Layered H3K4Me3
H3K4Me3 Mark (Often Found Near Promoters) on 7 cell lines from ENCODE
Layered H3K27Ac
H3K27Ac Mark (Often Found Near Active Regulatory Elements) on 7 cell lines from ENCODE
DNase Clusters
DNaseI Hypersensitivity Clusters in 125 cell types from ENCODE (V3)
Txn Factor ChIP
Transcription Factor ChIP-seq (161 factors) from ENCODE with Factorbook Motifs
Histone Modifications by ChIP-seq from ENCODE/Broad Institute
NHLF H3K4m1
NHLF H3K4m3
NHLF H3K9ac
NHLF H3K27ac
A549 DEX H3K4m1
A549 DEX H3K4m3
A549 DEX H3K27ac
A549 EtOH H3K4m1
A549 EtOH H3K4m3
A549 EtOH H3K9ac
A549 EtOH H3K27ac

Figure S2. Regulatory regions around 4 identified SNPs from ENCODE (Encyclopedia of DNA Elements).

(A) rs670343 at 2p23.3 was situated within the enhancer element (H3K4Me1 histone mark) on NHLF and A549 cell lines.

(B) rs9309336 at 2p15 was situated within the enhancer element (H3K27Ac histone mark) and fell into the promoter element (H3K4Me3 mark) on NHLF and A549 cell lines.

(C) rs17160062 at 1q21.1 located in the chromatin region with transcription factor binding signals by ChIP-seq.

(D) rs9252 at 17q21.2 located in the peak of H3K4Me1 chromatin modification (NHLF and A549 cell lines) and the region enriched of DNase Hypersensitivity clusters and transcription binding site.
